# Ten Simple Rules for Getting Published 

**DOI:** 10.1371/journal.pcbi.0010057

**Published:** 2005-10-28

**Authors:** Philip E Bourne

The student council (http://www.iscbsc.org/) of the International Society for Computational Biology asked me to present my thoughts on getting published in the field of computational biology at the Intelligent Systems in Molecular Biology conference held in Detroit in late June of 2005. Close to 200 bright young souls (and a few not so young) crammed into a small room for what proved to be a wonderful interchange among a group of whom approximately one-half had yet to publish their first paper. The advice I gave that day I have modified and present as ten rules for getting published.

## Rule 1: Read many papers, and learn from both the good and the bad work of others.

It is never too early to become a critic. Journal clubs, where you critique a paper as a group, are excellent for having this kind of dialogue. Reading at least two papers a day in detail (not just in your area of research) and thinking about their quality will also help. Being well read has another potential major benefit—it facilitates a more objective view of one's own work. It is too easy after many late nights spent in front of a computer screen and/or laboratory bench to convince yourself that your work is the best invention since sliced bread. More than likely it is not, and your mentor is prone to falling into the same trap, hence rule 2.

## Rule 2: The more objective you can be about your work, the better that work will ultimately become.

Alas, some scientists will never be objective about their own work, and will never make the best scientists—learn objectivity early, the editors and reviewers have.

## Rule 3: Good editors and reviewers will be objective about your work.

The quality of the editorial board is an early indicator of the review process. Look at the masthead of the journal in which you plan to publish. Outstanding editors demand and get outstanding reviews. Put your energy into improving the quality of the manuscript *before submission*. Ideally, the reviews will improve your paper. But they will not get to imparting that advice if there are fundamental flaws.

## Rule 4: If you do not write well in the English language, take lessons early; it will be invaluable later.

This is not just about grammar, but more importantly comprehension. The best papers are those in which complex ideas are expressed in a way that those who are less than immersed in the field can understand. Have you noticed that the most renowned scientists often give the most logical and simply stated yet stimulating lectures? This extends to their written work as well. Note that writing clearly is valuable, even if your ultimate career does not hinge on producing good scientific papers in English language journals. Submitted papers that are not clearly written in good English, unless the science is truly outstanding, are often rejected or at best slow to publish since they require extensive copyediting.

## Rule 5: Learn to live with rejection.

A failure to be objective can make rejection harder to take, and you will be rejected. Scientific careers are full of rejection, even for the best scientists. The correct response to a paper being rejected or requiring major revision is to listen to the reviewers and respond in an objective, not subjective, manner. Reviews reflect how your paper is being judged—learn to live with it. If reviewers are unanimous about the poor quality of the paper, move on—in virtually all cases, they are right. If they request a major revision, do it and address every point they raise both in your cover letter and through obvious revisions to the text. Multiple rounds of revision are painful for all those concerned and slow the publishing process.

## Rule 6: The ingredients of good science are obvious—novelty of research topic, comprehensive coverage of the relevant literature, good data, good analysis including strong statistical support, and a thought-provoking discussion. The ingredients of good science reporting are obvious—good organization, the appropriate use of tables and figures, the right length, writing to the intended audience—do not ignore the obvious.

Be objective about these ingredients when you review the first draft, and do not rely on your mentor. Get a candid opinion by having the paper read by colleagues without a vested interest in the work, including those not directly involved in the topic area.

## Rule 7: Start writing the paper the day you have the idea of what questions to pursue.

Some would argue that this places too much emphasis on publishing, but it could also be argued that it helps define scope and facilitates hypothesis-driven science. The temptation of novice authors is to try to include everything they know in a paper. Your thesis is/was your kitchen sink. Your papers should be concise, and impart as much information as possible in the least number of words. Be familiar with the guide to authors and follow it, the editors and reviewers do. Maintain a good bibliographic database as you go, and read the papers in it.

## Rule 8: Become a reviewer early in your career.

Reviewing other papers will help you write better papers. To start, work with your mentors; have them give you papers they are reviewing and do the first cut at the review (most mentors will be happy to do this). Then, go through the final review that gets sent in by your mentor, and where allowed, as is true of this journal, look at the reviews others have written. This will provide an important perspective on the quality of your reviews and, hopefully, allow you to see your own work in a more objective way. You will also come to understand the review process and the quality of reviews, which is an important ingredient in deciding where to send your paper.

## Rule 9: Decide early on where to try to publish your paper.

This will define the form and level of detail and assumed novelty of the work you are doing. Many journals have a presubmission enquiry system available—use it. Even before the paper is written, get a sense of the novelty of the work, and whether a specific journal will be interested.

## Rule 10: Quality is everything.

It is better to publish one paper in a quality journal than multiple papers in lesser journals. Increasingly, it is harder to hide the impact of your papers; tools like Google Scholar and the ISI Web of Science are being used by tenure committees and employers to define metrics for the quality of your work. It used to be that just the journal name was used as a metric. In the digital world, everyone knows if a paper has little impact. Try to publish in journals that have high impact factors; chances are your paper will have high impact, too, if accepted.

When you are long gone, your scientific legacy is, in large part, the literature you left behind and the impact it represents. I hope these ten simple rules can help you leave behind something future generations of scientists will admire. 

